# Accuracy of intraoperative aberrometry versus modern preoperative methods in post-myopic laser vision correction eyes undergoing cataract surgery with capsular tension ring placement

**DOI:** 10.1007/s00417-023-06327-3

**Published:** 2023-12-14

**Authors:** Allison J. Chen, Christopher P. Long, Tianlun Lu, Kevin J. Garff, Christopher W. Heichel

**Affiliations:** 1 Shiley Eye Institute, Division of Cornea, Cataract and Refractive Surgery, Viterbi Family Department of Ophthalmology, UC San Diego, 9415 Campus Point Drive, MC0946, La Jolla, CA 92093 USA; 2 USC Roski Eye Institute, Keck School of Medicine, Los Angeles, CA USA

**Keywords:** Intraoperative wavefront aberrometry, ORA, Laser vision correction, LASIK, PRK, Capsular tension ring

## Abstract

**Purpose:**

To assess the accuracy of intraoperative wavefront aberrometry (IWA) versus modern intraocular lens formulas in post-myopic laser vision correction (LVC) patients undergoing cataract surgery with capsular tension ring placement.

**Methods:**

This is a retrospective chart review conducted at an academic outpatient center. All post-myopic LVC eyes undergoing cataract surgery with IWA from a single surgeon from 05/2017 to 12/2019 were included. All patients received a capsular tension ring (CTR). Mean numerical error (MNE), median numerical error (MedNE), and percentages of prediction error within 0.50D, 0.75D, and 1.00D were calculated for the above formulas.

**Results:**

Twenty-seven post-myopic LVC eyes from 18 patients were included. In post-myopic LVC, MNE with Optiwave Refractive Analysis (ORA), Barrett True K (BTK), Haigis, Haigis-L, Shammas, SRK/T, Hill-RBF v3.0, and W-K AL-adjusted Holladay 1 were + 0.224, − 0.094, + 0.193, − 0.231, − 0.372, + 1.013, + 0.860, and + 0.630 (*F* = 8.49, *p* < 0.001). MedNE were + 0.125, − 0.145, + 0.175, + 0.333, + 0.333, + 1.100, + 0.880, and + 0.765 (*F* = 7.89, *p* < 0.001), respectively. BTK provided improved accuracy in both MNE (*p* < 0.001) and MedNE (*p* = .033) when compared to ORA in pairwise analysis. If the ORA vs. BTK-suggested IOL power were routinely selected, 30% and 15% of eyes would have projected hyperopic outcomes, respectively (*p* = 0.09).

**Conclusions:**

Our study suggests that in post-myopic LVC eyes undergoing cataract surgery with CTRs, BTK performed more accurately than ORA with regard to accuracy and yielded a lower percentage of eyes with hyperopic outcomes. Haigis, Haigis-L, and Shammas yielded similar results to ORA with regard to accuracy and percentage of eyes with hyperopic outcomes. On average, Shammas and Haigis-L suggested IOLs that would yield outcomes more myopic than expected when compared to BTK.

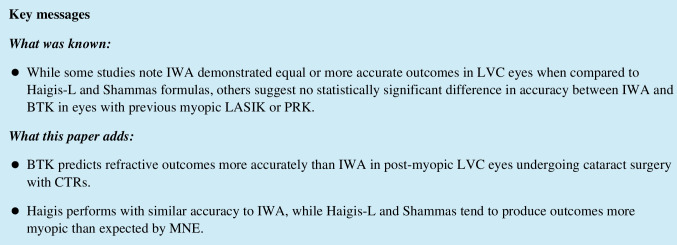

**Supplementary Information:**

The online version contains supplementary material available at 10.1007/s00417-023-06327-3.

## Introduction

Modern intraocular lens (IOL) prediction methods (Hill-RBF v3.0, Barrett Universal II/True K, intraoperative wavefront aberrometry) have demonstrated improved accuracy in predicting refractive outcomes over older generation biometric formulas [1, 2]. However, IOL selection in patients who have had prior laser vision correction (LVC) surgery continues to remain a challenge as older generation IOL formulas do not take into account the alteration of the cornea’s natural topography when utilizing measured keratometry inputs in post LVC eyes.

Since the first LVC procedure (photorefractive keratectomy) performed in 1988, there have since been 10 million Americans who have had LASIK or PRK, with approximately 700,000 to > 1 million surgeries performed yearly. As the LVC patient population approaches the age of cataract surgery, the demand for accurate IOL predictions in prior LVC eyes continues to grow. Currently, studies suggest lower IOL power prediction accuracy in these eyes, with a satisfactory outcome of ± 0.5 D of the target in less than 80% of cases [3]. Previous studies have suggested that intraoperative wavefront aberrometry (IWA) has demonstrated improvement in refractive outcomes when compared to Haigis-L, Shammas Method, Masket Formulae, SRK/T, and the ASCRS website [4–6]. More recent studies have focused on comparing IWA to newer formulas tailored toward predicting IOL outcomes in post-LVC eyes, such as Barrett True K (BTK). Some have demonstrated IWA to be superior to BTK [7], but the difference is small and other studies suggest the results are similar between the two [8–11]. Although previous studies have evaluated other next generation formulas such as Haigis and AL-adjusted Holladay 1 compared to IWA in normal eyes [12, 13], few have compared these additional formulas in the setting of post LVC eyes [14], and none have included Hill-Radial Basis Function Version 3.0 (Hill-RBF v3.0) in their analysis. Even further, there have been no studies comparing IWA to any of these formulas in cases with capsular tension rings, which have demonstrated lower variance in refractive outcomes than in contralateral eyes without a CTR [15]. To this end, our study assesses the accuracy of IWA and modern IOL formulas in post-refractive surgery patients undergoing cataract surgery with capsular tension rings.

## Methods

Institutional review board (IRB) approval was obtained for the study. The study adhered to the tenets of the Declaration of Helsinki. A retrospective chart review was performed for all patients with prior LVC (LASIK or PRK) undergoing cataract surgery with IWA (Optiwave Refractive Analysis system, ORA) by the same surgeon from 05/2017 to 12/2019. All surgeries were performed at the institution’s ambulatory surgery center (UC San Diego Shiley Eye Institute, La Jolla, CA USA). The same surgical protocol was used for all recruited patients; all surgeries were performed via a 2.4-mm main incision size and without sutures or intraoperative complications. Eyes were ineligible for study if they had previous ocular surgery, trauma, or vision-limiting retinal or posterior segment disease. Of note, a capsular tension ring was placed in all eyes.

A power calculation was performed to determine the number of eyes needed to detect an effect size of 0.250 difference in refractive prediction error between formulas at a significance level of α = 0.05 and β = 0.80. The standard deviation used for the power calculation was derived from a previous study utilizing similar formulas [2]. The estimated required sample size was 24 eyes, and it was ensured that the total number of eyes within the study’s date range exceeded our estimated required sample size.

Data gathered included the chosen IOL type, IOL diopter power, target refractive aim during the pre-operative visit, and refractive predictions (in diopters) for the chosen IOL as predicted by (1) BTK (no history), (2) Holladay 1 without modification, (3) AL-optimized Holladay 1, (4) Hill-RBF v3.0, (5) Haigis, (6) Haigis-L*, (7) Shammas*, (8) SRK/T, and (9) IWA in the aphakic state. The equation for the optimized AL was performed for eyes with AL greater than 25.0 mm [16] and is as follows: AL_IOLmaster500_ × 0.8289 + 4.2663. The W-K optimized AL was manually substituted back into the IOL Master 500 to obtain this method’s refractive prediction, as described by Wang et al. [16]. The same IOL Master 500 was used in all cases. Eyes with AL < 25.0 mm did not undergo W-K AL adjustment. *Of note, BTK was calculated using the direct formula link on the APACRS website. Further, since our IOLmaster could not directly calculate the predicted refraction for the chosen IOL for the Haigis-L and Shammas formulas, Haigis-L and Shammas refractive predictions for the chosen IOL were back-calculated from their respective formula-suggested IOL power from the ASCRS post-refractive website [17]. The recommended IOL diopter power was translated into a projected MRx by the below “back-calculated” method, using Shammas as an example:

MRx Prediction_Shammas_ for IOL power chosen = Postoperative month 1 spherical equivalent + (Shammas-suggested IOL power – actual IOL power used) * (2/3). The “2/3” multiplier was utilized to translate the IOL plane diopter power into the MRx plane refraction. The Shammas and Haigis-L IOL powers suggested from the online ASCRS calculator were rounded to the nearest 0.5D for formula purposes.

Postoperative manifest refraction was obtained and recorded by an in-clinic optometrist at the patient’s post-operative visit month 1 visit (no fewer than 28 days and no more than 45 days after surgery). The same optometrist aided in all postoperative refractions. Primary outcome measures included the difference between the predicted refraction and the actual postoperative spherical equivalent (numerical error), the median numerical error, and the proportion of patients within ± 0.50D, ± 0.75D, and ± 1.00D for each prediction method.

### Statistical analysis

Mean numerical error (MNE), median numerical error (MedNE), and percent of eyes within ± 0.50D, ± 0.75D, and ± 1.00D were calculated for post-myopic LVC eyes. Mean numerical error was calculated as the mean of patients’ actual postoperative spherical equivalent (SE) minus the predicted SE. Median numerical error was calculated as the median of the differences between the actual postoperative SE and the predicted SE. A hyperopic outcome was defined as any outcome that was hyperopic relative to the predicted SE and was not necessarily a positive SE.

The MNE and MedNE in the 9 groups were compared using repeated-measures analysis of variance (ANOVA) with a significance level set at α = 0.05. Median numerical errors were compared using repeated-measures rank ANOVA. Post hoc pairwise analyses utilizing two-sided *t*-tests with Tukey HSD corrections for multiple pairwise analyses were performed to identify statistically significant differences between IOL prediction methods for MNE, the percentage of eyes within 0.5D of target spherical equivalent, and percentage of eyes with outcomes more hyperopic than predicted.

Further calculations were performed to project the percentage of eyes that would have had projected hyperopic outcomes (this time defined as a refraction with SE > 0) if the ORA versus BTK- “suggested” IOL power were always selected. The respective suggested IOL powers were based on the target aim which was pre-defined in all eyes, and was bolded by the respective software.

If the chosen IOL was different than the suggested IOL, the projected refractive outcomes were “back-calculated” by adjusting the actual postoperative month 1 manifest refraction based on the optical translation of a 1.5D change in the IOL plane correlating to a 1.0D change in refraction at the retinal plane. For purposes of explanation, if a postoperative month 1 refractive outcome (SE) was − 0.50 sph, and the ORA suggested a lens power that was 0.5D lower than the chosen IOL, then the projected refractive outcome (SE) for the ORA-suggested-lens would be − 0.50 + 0.333 =  − 0.167, which in this scenario would *not* be considered a hyperopic outcome given that − 0.167 is a negative value. All statistical analysis was performed utilizing StataSE (College Station, TX) and RStudio v1.2 (Boston, MA).

## Results

Twenty-seven post-myopic LVC eyes from 18 patients were identified. Twenty-one received monofocal (*n* = 19) or non-toric multifocal (*n* = 2) lenses, and 6 received toric lenses. The mean IOL power (D) implanted was 19.9D (± 0.3D StErr). Outcome comparisons across all 9 methods—MNE, MedNE, percentage of eyes within ± 0.50D, ± 0.75D, and ± 1.00D and with hyperopic outcomes—are listed in Table 1.

**Table 1:** Comparison of the 9 calculation methods

All IOLs (monofocal, MF, toric) *N* = 27	MNE	STERR	MedNE (Q1, Q3)	Percent within ± 0.50D	Percent within ± 0.75D	Percent within ± 1.00D	Hyperopic (%)*
Wavefront aberrometer	+ 0.224	0.119	+ 0.125 (− 0.077, 0.550)	62.9	70.3	85.2	70.3
Barrett True K	− 0.094	0.114	− 0.145 (− 0.377,0.155)	74.1	88.9	88.9	33.3
Haigis	+ 0.193	0.135	+ 0.175 (− 0.120, 0.480)	62.9	77.8	88.9	59.2
Haigis-L	− 0.231	0.162	− 0.333 (− 0.666, 0)	61.5	84.6	88.5	57.7
Shammas	− 0.372	0.165	− 0.333(− 0.666, 0)	46.2	73.1	88.5	65.3
SRK/T	+ 1.013	0.131	+ 1.100 (0.612, 1.475)	18.5	25.9	44.4	92.6
Holladay 1	+ 0.920	0.143	+ 1.000 (0.467, 1.43)	18.5	37.0	51.9	88.9
AL-optimized Holladay 1	+ 0.630	0.120	+ 0.765 (0.342, 0.912)	33.3	44.4	81.5	81.5
Hill RBF	+ 0.860	0.127	+ 0.880 (0.490, 1.24)	25.9	37.0	55.6	92.6
*p*-value	< 0.001		< 0.001	< 0.001	< 0.001	< 0.001	< 0.001

Monofocal/MF IOLs only (*n* = 21)	MNE	STERR	MedNE (Q1, Q3)	Percent within ± 0.50D	Percent within ± 0.75D	Percent within ± 1.00D	Hyperopic (%)*
Wavefront aberrometer	+ 0.185	0.121	+ 0.08 (− 0.005, 0.310)	66.7	76.2	90.4	71.4
Barrett True K	− 0.018	0.121	− 0.145 (− 0.290, 0.170)	81.0	90.5	90.5	33.3
Haigis	+ 0.221	0.139	+ 0.175 (− 0.120, 0.345)	76.2	90.5	95.2	57.1
Haigis-L	− 0.150	0.117	− 0.167 (− 0.500, 0)	66.7	95.2	95.2	9.5
Shammas	− 0.200	0.127	− 0.333 (− 0.667, 0)	61.9	85.7	95.2	14.3
SRK/T	+ 1.080	0.112	+ 1.10 (0.815, 1.430)	19.0	23.8	42.9	100.0
Holladay 1	+ 0.987	0.129	+ 1.00 (0.605, 1.34)	19.0	33.3	52.4	95.2
AL-optimized Holladay 1	+ 0.725	0.111	+ 0.765 (0.455, 0.886)	38.1	47.6	85.7	90.5
Hill RBF	+ 0.930	0.132	+ 0.880 (0.510, 1.210)	23.8	38.1	57.1	95.2
*p*-value	< 0.001		< 0.001	< 0.001	< 0.001	< 0.001	< 0.001

*Hyperopic in this column is defined as a more positive (SE) outcome than predicted

*IOL* intraocular lens, *MedNE* median numerical error, *MF* multifocal, *MNE* mean numerical error, *STERR* standard error

Pairwise comparisons of MNE among the 9 methods are demonstrated in Table 2. BTK produced significantly lower MNE when compared to IWA (*p* < 0.001) and Haigis (*p* < 0.001). IWA produced MNEs similar to Haigis (*p* = 0.754), Haigis-L (*p* = 0.995), and Shammas (0.530). BTK also demonstrated significantly improved accuracy when compared to IWA with regard to MedNE (*p* = 0.033).

**Table 2:** The *p*-values for pairwise comparisons of MNE between IOL calculation methods

All IOLs (monofocal, MF, toric)	IWA	Barrett True K	Haigis	Haigis-L	Shammas	SRK/T	Holladay 1	AL-optimized Holladay 1
Barrett True K	< 0.001*	–	–	–	–	–	–	–
Haigis	0.754	< 0.001*	–	–	–	–	–	–
Haigis-L	0.995	0.158	0.842	–	–	–	–	–
Shammas	0.530	0.058	0.470	0.013*	–	–	–	–
SRK/T	< 0.001*	< 0.001*	< 0.001*	0.003*	0.014*	–	–	–
Holladay 1	< 0.001*	< 0.001*	< 0.001*	0.014*	0.046*	0.004*	–	–
AL-optimized Holladay 1	< 0.001*	< 0.001*	< 0.001*	0.040*	0.148	< 0.001*	0.003*	–
Hill RBF	< 0.001*	< 0.001*	< 0.001*	0.009*	0.043*	0.0457*	0.477	0.027*

*AL* axial length, *IWA* intraoperative wavefront aberrometry, *MF* multifocal, *IOL* intraocular lens, *MNE* mean numerical error

*Statistically significant

**Fig. 1:**
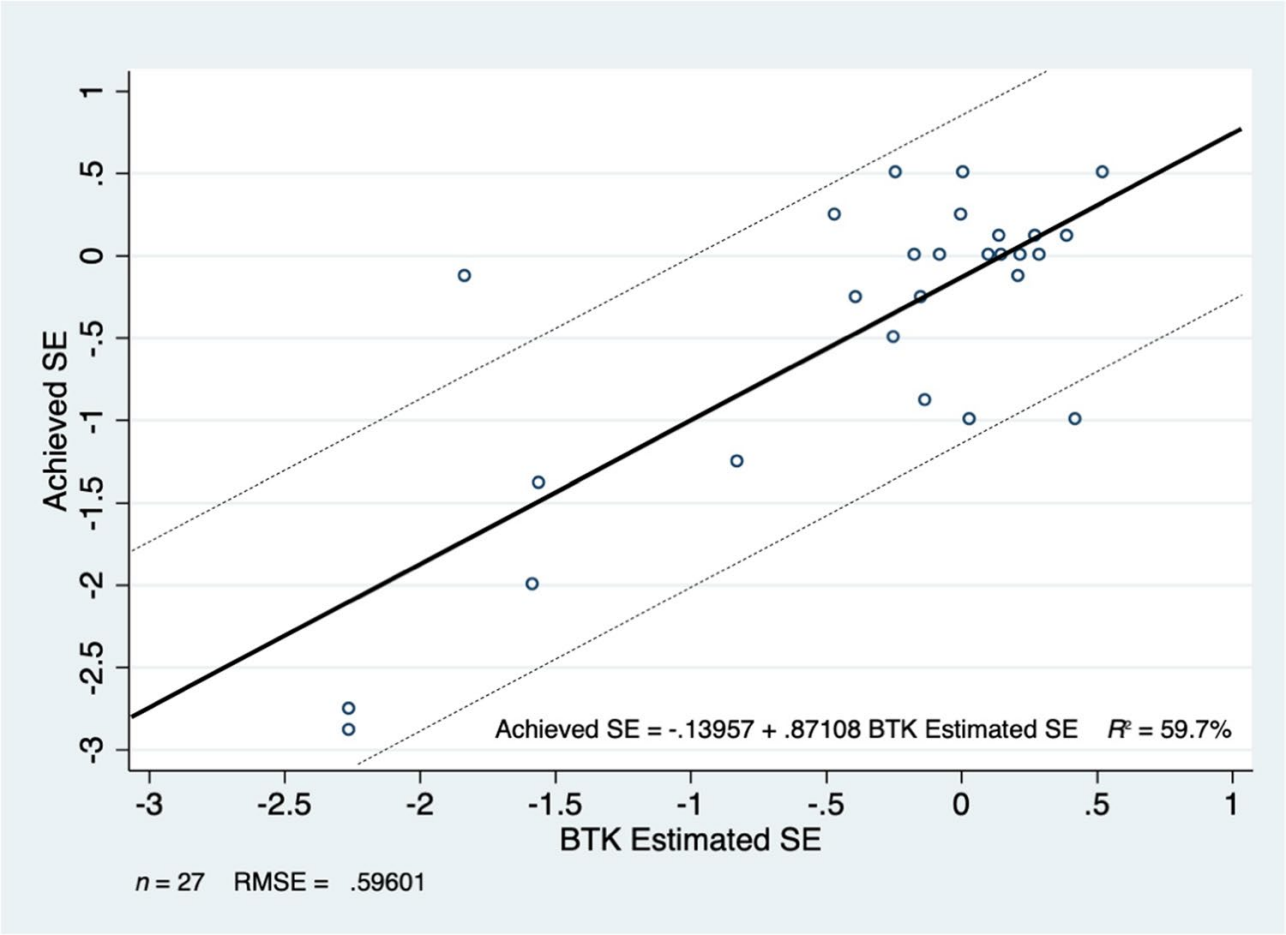
Barrett True K predicted SE versus post-operative achieved SE. Dark line illustrates line of best fit, and dashed lines indicate ± 1D

**Fig. 2:**
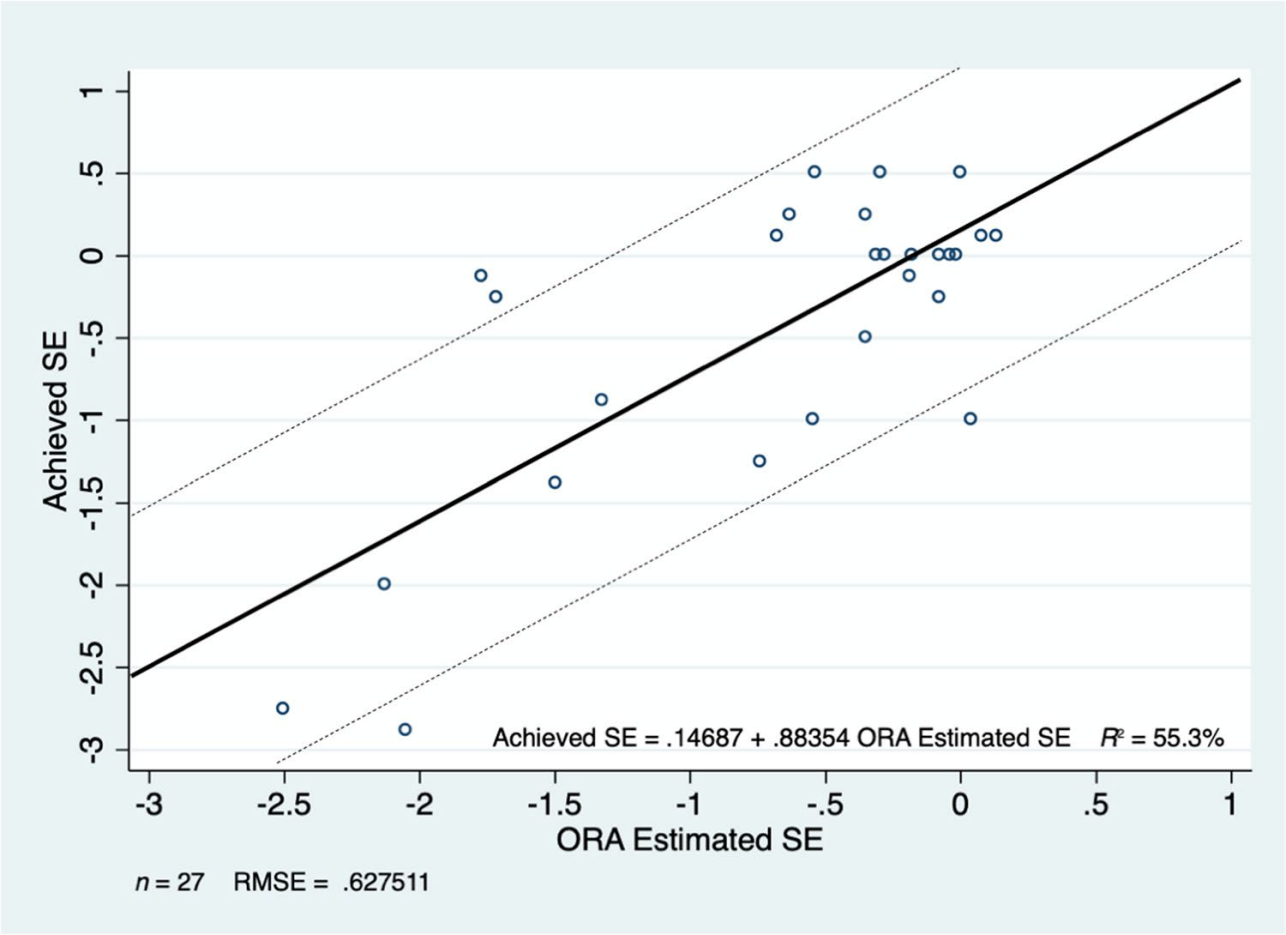
ORA (IWA) predicted SE versus post-operative achieved SE. Dark line illustrates line of best fit, and dashed lines indicate ± 1D

Predicted versus achieved SE for BTK and IWA are numerically and graphically illustrated in Supplementary Appendix, and Figs. 1 and 2.

BTK, IWA, Haigis, and Haigis-L performed similarly with regards to predicting a SE within ± 0.5D of the actual postoperative SE (Table 3). These methods performed significantly better than AL-optimized Holladay 1, Holladay 1, Hill-RBF v3.0, and SRK/T. Though Shammas produced similar results as IWA (*p* = 0.202) and Haigis (0.202), both BTK (*p* = 0.043) and Haigis-L (*p* = 0.043) demonstrated significantly higher percent of eyes within 0.5D of target SE than Shammas.

**Table 3:** The *p*-values for pairwise comparisons of percentage of eyes within 0.5D of target SE for each IOL calculation method

All IOLs (monofocal, MF, toric) *N* = 27	IWA	Barrett True K	Haigis	Haigis-L	Shammas	SRK/T	Holladay 1	AL-optimized Holladay 1
Barrett True K	0.264	−	−	−	−	−	−	−
Haigis	1.00	0.184	−	−	−	−	−	−
Haigis-L	0.769	0.255	0.769	−	−	−	−	−
Shammas	0.202	0.043**	0.202	0.043*	−	−	−	−
SRK/T	0.001*	< 0.001*	0.001*	0.002*	0.050	−	−	−
Holladay 1	0.002*	< 0.001*	< 0.001*	0.001*	0.032*	1.00	−	−
AL-optimized Holladay 1	0.043*	0.008*	0.029*	0.069	0.449	0.043*	0.043*	−
Hill RBF	0.015*	0.001*	0.005*	0.017*	0.202	0.326	0.327	0.161

*AL* axial length, *IWA* intraoperative wavefront aberrometry, *MF* multifocal, *IOL* intraocular lens, *MNE* mean numerical error, *SE* spherical equivalent

*Statistically significant

The nine methods differed significantly in the proportion of patients with hyperopic outcomes (*p* < 0.001) as defined by outcomes more hyperopic than predicted (and not necessarily a positive SE). BTK significantly reduced hyperopic outcomes compared to IWA, Haigis, Shammas, AL-optimized Holladay 1, Holladay 1, SRK/T, and Hill-RBF v3.0 (Table 4). BTK and Haigis L did not differ significantly in percentage of patients with hyperopic outcomes. IWA did not differ from Haigis, Haigis-L, Shammas, or AL-optimized Holladay 1 in reducing hyperopic outcomes but was superior to SRK/T and Hill-RBF v3.0.

**Table 4:** The *p*-values for pairwise comparisons of IOL calculation methods regarding percentage of eyes with hyperopic outcomes^a^

All IOLs (monofocal, MF, toric) *N* = 27	IWA	Barrett True K	Haigis	Haigis-L	Shammas	SRK/T	Holladay 1	AL-optimized Holladay 1
Barrett True K	< 0.001*	–	–	–	–	–	–	–
Haigis	0.327	0.0165*	–	–	–	–	–	–
Haigis-L	0.265	0.089	1.000	–	–	–	–	–
Shammas	0.769	0.047*	0.626	0.424	–	–	–	–
SRK/T	0.031*	< 0.0001*	0.001*	0.004*	0.017*	–	–	–
Holladay 1	0.0571	< 0.0001*	0.003*	0.009*	0.031*	0.326	–	–
AL-optimized Holladay 1	0.103	< 0.0001*	0.006*	0.016*	0.096	0.161	0.326	–
Hill RBF	0.011*	< 0.0001*	0.001*	0.001*	0.005*	1.000	0.326	0.161

*AL* axial length, *IOL* intraocular lens, *IWA* intraoperative wavefront aberrometry, *MF* multifocal

^a^Hyperopic is defined as resulting in a more positive (SE) outcome than predicted

*Statistically significant

If the IWA-suggested IOLs were always selected, 29.6% (8/27 eyes) would have had hyperopic outcomes (this time defined as a SE > 0), whereas if the BTK suggested IOL were always selected, 14.8% (4/27) of eyes would have had hyperopic outcomes (*p* = 0.09). The IWA and BTK-suggested IOL powers differed in 55.6% of eyes (15/27), 13 in which BTK suggested a higher IOL diopter power than IWA, and 2 in which BTK suggested a lower IOL power. Across all post-myopic LVC eyes, the mean of the difference between the IOL-suggested powers between BTK and IWA was − 0.5D (St Err: 0.16D) (IWA 19.9D vs BTK 20.4D, *p* < 0.005), suggesting that overall, IWA suggested a lens that was 0.5D significantly lower than the BTK-suggested IOL.

## Discussion

With increasing patient expectations for accurate refractive outcomes after cataract surgery, ophthalmologists have made strides toward accurate IOL selection. Specifically in post-LVC eyes which have altered corneal topographies, the hope is that IWA can provide patient specific, real-time measurements and provide precise predictions during surgery. However, previous authors have illustrated the multiple challenges hindering IWA measurements, including IOP levels, lid-speculum-induced pressure or astigmatism, and the sensitivity and ranges of the wavefront sensors themselves [18].

The accuracy of the ORA system, one of the two intraoperative wavefront sensors available in the USA, has been previously evaluated in post-LVC eyes and suggested to be superior to Haigis-L and Shammas IOL [4]. However, recent research has called this superiority into question. For example, in Fram et al.’s study, there was not a statistically significant difference in mean or median absolute errors among ORA, the Masket regression formula, Fourier-Domain OCT-Based Formula (Optovue), or Haigis-L when evaluating LVC eyes [5].

More recently, the superiority of IWA has been challenged by next-generation formulas such as BTK, Haigis, AL-adjusted Holladay 1, and Hill-RBF. Previous studies conducted in normal eyes by Sakai et al. and Geenwood et al. each compared IWA with these formulas and suggest use of these preoperative formula produces results equal to or superior to IWA [12, 13].

However, further research has been needed to compare the accuracy of outcomes between these formulas and IWA in post-refractive eyes. In Christopher et al.’s study [8], there was not a statistically significant difference in mean absolute error between ORA and BTK. Curado et al. compared IWA with BTK, Haigis, and Holladay formulas, demonstrating there is no significant difference between median absolute error and mean absolute error in these groups.

In our study, we found that BTK yielded significantly more accurate MNE and MedNE than IWA. Similar to Fram et al.’s study [5], there was no difference in accuracy between IWA and Haigis-L, and we additionally did not find a difference in accuracy between IWA and Haigis/Shammas.

To our knowledge, this is one of the first studies to evaluate IWA in comparison to BTK, Haigis, AL-optimized Holladay 1, and Hill-RBF v3.0 in post-myopic LVC eyes receiving CTRs. In our study, we found that BTK demonstrated a statistically significant reduction in MNE and percentage of hyperopic outcomes when compared to IWA. Our results are corroborated by our finding that on average IWA was “suggesting” a lens approximately 0.5D statistically significantly smaller in magnitude than the BTK-suggested lens. This difference may imply that the IWA is not incorporating an appropriately “flat enough” cornea in eyes with post-myopic ablations, or that the BTK method is able to more accurately incorporate such information. Our findings may assist surgeons utilizing IWA to lean toward the higher diopter lens if straddling a lens choice in the operating room to minimize the chances of a hyperopic surprise.

Our results demonstrated that 62.9% of eyes were within ± 0.50D of the IWA prediction, which is lower than the currently highest reported percentage of 74% by Fram et al. [5]. However, our results suggest that 74.1% of eyes were within ± 0.50D of the BTK prediction, which is comparable to the currently highest published percentage of post-myopic LASIK eyes falling within ± 0.50D of preoperative or intraoperative predictions [5, 8, 19].

Although it is difficult to determine why BTK outperforms IWA, a potential explanation could be that IWA is measuring eyes that have been altered by drops, surgery with corneal edema, and other factors that affect the quality of ocular surface. Thus, the ocular surface can vary widely among patients and contribute to variability in measurement quality when using IWA. Secondly, BTK may potentially have a more accurate algorithm for power calculations, though both methods are a black box and it is not publically known as to how they predict the estimated lens position.

This study has several limitations. First, it is a retrospective analysis. Though difficult to implement due to practice setting, a prospective study randomizing eyes to IOL selection using BTK or IWA would be more robust. Secondly, all eyes in our study received a capsular tension ring (CTR). This may theoretically affect the final effective lens position (ELP); a previous study suggests that eyes receiving CTRs demonstrated lower variance in refractive outcomes than in contralateral eyes without a CTR [15]. Thirdly, there was a large variety of IOL types implanted, including monofocal, toric, and multifocal which may introduce unmeasured factors. However, when excluding torics, similar results were found which supports the robustness of our results. Fourthly, this study comprises fewer eyes than some of the more recent studies [8, 11] which may limit the statistical power of some formula comparisons—however, notable results were still unearthed and contribute to the current body of literature. Lastly, the performance of BTK was assessed without inputting historical data, and our results may not be generalizable to cases in which historical data is inputted.

In conclusion, our study suggests that in post-myopic LVC eyes, Barrett True-K performs more effectively than ORA wavefront aberrometry with regard to accuracy and decreasing the percentage of eyes with hyperopic outcomes. Our outcomes suggest that BTK predictions should be incorporated in the process of lens selection even in places which do have access to IWA, and that if straddling a lens power choice in the operating room, leaning toward the higher IOL power when utilizing IWA in the aphakic mode may yield a more accurate outcome, even if the intraoperative suggested lens may be a lower power. ORA was found to be as effective as Haigis, Haigis-L, and Shammas, and these additional pre-operative biometric formulas can be helpful in determining and cross-validating lens selection, particularly in decreasing the risk of hyperopic outcomes. Haigis and Haigis-L were found to be comparable with regard to accuracy and percentage of eyes with hyperopic outcomes. Of note, Haigis-L and Shammas tended to produce outcomes more myopic than BTK by MNE estimates, though not statistically significant. Further research is needed to identify methods to improve our predictive accuracy in post-laser vision correction eyes and to evaluate our current IOL selection methods in additional populations, such as in eyes without CTRs, across multiple surgeons, and in post-hyperopic LVC.

### Supplementary Information

Below is the link to the electronic supplementary material.

ESM 1 (DOCX 16.6 KB)
