# Supplementary material for: Accuracy of intraoperative aberrometry versus modern preoperative methods in post-myopic laser vision correction eyes undergoing cataract surgery with capsular tension ring placement
Source: Graefes Arch Clin Exp Ophthalmol. 2023 Dec 14;262(5):1545–52. doi: 10.1007/s00417-023-06327-3 (PMC11031443; doi:10.1007/s00417-023-06327-3)
Supplement: Supplementary file 1 — ESM 1 (DOCX 16.6 KB) [file 417_2023_6327_MOESM1_ESM.docx]

|  |  |  |  |
| --- | --- | --- | --- |
| Eye # | Achieved SE | BTK Estimated SE | ORA Estimated SE |
| 1 | 0.125 | 0.14 | 0.13 |
| 2 | -0.125 | -1.83 | -1.77 |
| 3 | -0.125 | 0.21 | -0.19 |
| 4 | -0.250 | -0.39 | -1.72 |
| 5 | -2.875 | -2.26 | -2.05 |
| 6 | 0.250 | -0.47 | -0.63 |
| 7 | 0.125 | 0.27 | 0.08 |
| 8 | 0.000 | -0.17 | -0.28 |
| 9 | 0.500 | -0.24 | -0.30 |
| 10 | -2.000 | -1.58 | -2.13 |
| 11 | -1.000 | 0.03 | 0.04 |
| 12 | -1.000 | 0.42 | -0.55 |
| 13 | -0.875 | -0.13 | -1.33 |
| 14 | 0.000 | 0.15 | -0.08 |
| 15 | -2.750 | -2.26 | -2.50 |
| 16 | -1.250 | -0.83 | -0.74 |
| 17 | -0.250 | -0.15 | -0.08 |
| 18 | 0.500 | 0.01 | 0.00 |
| 19 | 0.500 | 0.52 | -0.54 |
| 20 | -1.375 | -1.56 | -1.50 |
| 21 | 0.000 | 0.10 | -0.04 |
| 22 | 0.125 | 0.39 | -0.68 |
| 23 | 0.000 | 0.22 | -0.18 |
| 24 | 0.250 | 0.00 | -0.35 |
| 25 | 0.000 | 0.29 | -0.02 |
| 26 | 0.000 | -0.08 | -0.31 |
| 27 | -0.500 | -0.25 | -0.35 |
|  |  |  |  |

Appendix 1. Achieved versus predicted spherical equivalence: Barrett True K and ORA

SE = spherical equivalent; BTK = Barrett True K
